# Efficient linkage mapping using exome capture and extreme QTL in schistosome parasites

**DOI:** 10.1186/1471-2164-15-617

**Published:** 2014-07-21

**Authors:** Frédéric D Chevalier, Claudia LL Valentim, Philip T LoVerde, Timothy JC Anderson

**Affiliations:** Department of Genetics, Texas Biomedical Research Institute, P.O. Box 760549, 78245 San Antonio, Texas USA; Departments of Biochemistry and Pathology, University of Texas Health Science Center, 78229-3900 San Antonio, Texas USA

**Keywords:** X-QTL, Exome capture, *Schistosoma mansoni*, Oxamniquine resistance, NGS

## Abstract

**Background:**

Identification of parasite genes that underlie traits such as drug resistance and host specificity is challenging using classical linkage mapping approaches. Extreme QTL (X-QTL) methods, originally developed by rodent malaria and yeast researchers, promise to increase the power and simplify logistics of linkage mapping in experimental crosses of schistosomes (or other helminth parasites), because many 1000s of progeny can be analysed, phenotyping is not required, and progeny pools rather than individuals are genotyped. We explored the utility of this method for mapping a drug resistance gene in the human parasitic fluke *Schistosoma mansoni.*

**Results:**

We staged a genetic cross between oxamniquine sensitive and resistant parasites, then between two F1 progeny, to generate multiple F2 progeny. One group of F2s infecting hamsters was treated with oxamniquine, while a second group was left untreated. We used exome capture to reduce the size of the genome (from 363 Mb to 15 Mb) and exomes from pooled F2 progeny (treated males, untreated males, treated females, untreated females) and the two parent parasites were sequenced to high read depth (mean = 95-366×) and allele frequencies at 14,489 variants compared. We observed dramatic enrichment of alleles from the resistant parent in a small region of chromosome 6 in drug-treated male and female pools (combined analysis:  = 11.07, *p* = 8.74 × 10^-29^). This region contains Smp_089320 a gene encoding a sulfotransferase recently implicated in oxamniquine resistance using classical linkage mapping methods.

**Conclusions:**

These results (a) demonstrate the utility of exome capture for generating reduced representation libraries in *Schistosoma mansoni*, and (b) provide proof-of-principle that X-QTL methods can be successfully applied to an important human helminth. The combination of these methods will simplify linkage analysis of biomedically or biologically important traits in this parasite.

**Electronic supplementary material:**

The online version of this article (doi:10.1186/1471-2164-15-617) contains supplementary material, which is available to authorized users.

## Background

**Figure 1:**
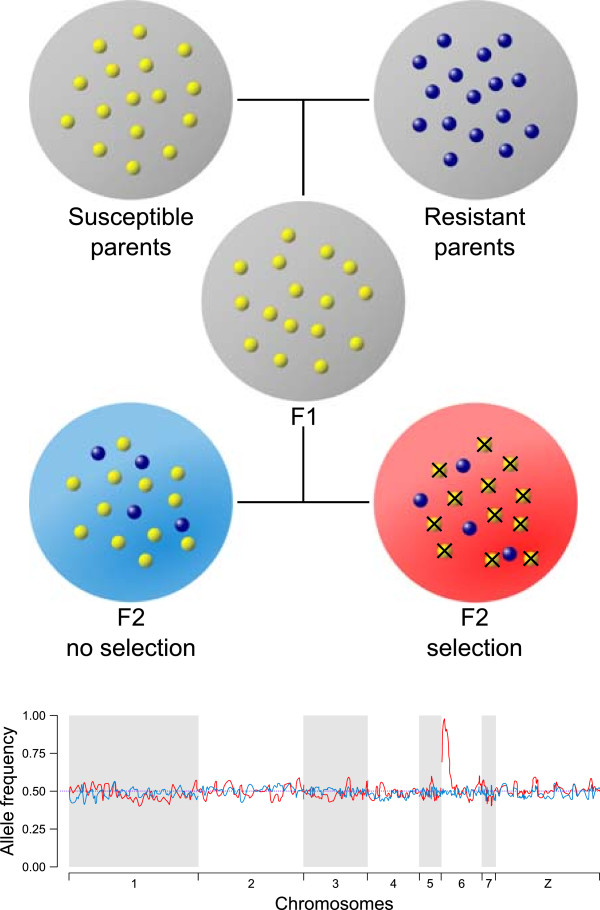
**Principle of the extreme-QTL (X-QTL) analysis.** X-QTL analysis can be performed on a simple Mendelian inherited trait (for instance drug susceptibility (dominant trait) and drug resistance (recessive trait)) using pools of individuals (grey circle). Individuals with susceptible phenotype (yellow balls) and resistant phenotype (dark blue balls) are crossed to generate F1 and F2 individuals. The F2 individuals are divided in two pools: one under no selection (without drug treatment, light blue circle) and another under selection (with drug treatment, red circle). The allele frequency is equal in unselected (light blue line) and selected (red line) pools across the genome, except in the genome region (chromosome 6 here) containing the critical genes that underlie the selected trait.

Classical methods for QTL identification involve examination of cosegregation of markers and trait loci in the parents and progeny of experimental genetic crosses or in extended pedigrees [1, 2]. However, this requires both phenotyping and genotyping of multiple individual progeny which is time consuming, labor intensive, expensive and places upper limits on statistical power [2]. Extreme QTL (X-QTL) methods provide a simple way to circumvent these disadvantages and are applicable to any selectable trait (Figure 1). In X-QTL, large groups of progeny are selected for the trait of interest and compared with control groups that are not exposed to selection. Selected and unselected pools are quantitatively genotyped to estimate frequencies of genetic markers across the genome. Selected and unselected pools show equal representation of frequency across the genome, except in genome regions containing the critical genes that underlie the selected trait. X-QTL methods are a development of bulk segregation methods, originally established by human and plant geneticists in the mid-1980s and the early 1990s [3–5], in which individual progeny with different phenotypes were pooled and genotyped. X-QTL methods were independently developed by researchers working on rodent malaria, where the method was referred to as linkage group selection [6–8] and was later used on yeast [9], where the term X-QTL was coined. We prefer the name X-QTL and have adopted this term throughout.

Here we validate the X-QTL approach for linkage analysis of a biomedically important trait (oxamniquine (OXA) resistance) in the human blood fluke *Schistosoma mansoni* for which the causative gene (Smp_089320 on chromosome 6) is now known [10]. Schistosomiasis, the second most important tropical parasitic disease after malaria [11], affects an estimated 187 million people across Africa, Asia, and South America, killing over 200,000 people per year and is caused by blood flukes of the genus *Schistosoma* [12–14]. Among the three major schistosome species of medical importance, *S. mansoni* infects over 67 million people in Africa, Middle East and South America [15]. The life cycle of this parasite includes a snail as intermediate host (*Biomphalaria* spp.) and humans as definitive host. However, the complete lifecycle can be easily maintained in the laboratory using snail and rodent hosts (Additional file 1: Figure S1) [16], and there is a rich literature describing heritable variation in a range of biomedically and biologically interesting traits such as drug resistance [17, 18], host specificity [19], and virulence [20]. The 364.5 Mb genome of *S. mansoni* has been sequenced [21], and with the aid of a 5 cM genetic map [22] 80% of the scaffolds have been assigned and ordered on chromosomes [23]. The genome is composed of 7 autosomes and one pair of ZW sex chromosomes and contains 10,852 genes. Forty percent of the genome is composed of repetitive elements [21, 23].

We have recently exploited the genome sequence and genetic map to identify, by classical linkage mapping, a QTL region underlying parasite resistance to oxamniquine, one of the two drugs used in the treatment of *S. mansoni* infection [10]. This is the first trait mapped in *S. mansoni* or in any human helminth infection and resulted in direct identification of the gene and mutations responsible for this trait. While classical genetic mapping is clearly feasible in this organism, it is extremely labor intensive. In the oxamniquine genetic cross, 2,856 individual snails were exposed to single larval stages to obtain 388 snails infected with F2 progeny, while measurement of resistance involved daily observation of worm death following drug exposure over a two week period for each of the F2 progeny genotypes. Hence mapping of OXA-resistance QTL required a concerted effort by three researchers over a two year period. We therefore sought to develop more efficient methods for linkage mapping in this parasite.

X-QTL methods require accurate measurement of genome wide allele frequencies within progeny pools. This has been done by pyrosequencing [24], microarray hybridization [25], and more recently by examining read depth of SNPs using next generation sequence data (Pool-seq) [26]. The genome of *S. mansoni* is relatively large (364.5 Mb), and comprises ~40% repetitive sequences, so we sought to use reduced representation sequencing to minimize cost and maximize read depth in F2 progeny pools. Several methods using restriction enzymes have been developed for performing reduced representation sequencing for complex genomes (e.g. restriction-site-associated DNA sequencing (RAD-seq), reduced-representation libraries (RRL)) [27]. We opted to use exome capture rather than restriction based methods for several reasons: (i) obtaining exome sequence data simplifies fine mapping of loci identified following initial QTL location, as most variants involved in phenotypic traits result from changes within coding sequences [28], (ii) we wanted to avoid sequencing repetitive regions that cannot be unambiguously aligned in a single genomic location (this is a particular concern for *S. mansoni* given the repeat content of this genome), (iii) polymorphisms within restriction sites have the potential to bias representation of parental alleles within progeny pools when using approaches such as RAD-seq [29]. This can potentially generate spurious enrichment of particular alleles within progeny pools. A second aim of this work was therefore to evaluate the efficiency of exome capture methods for *S. mansoni*.

## Results

### Genetic crosses

We staged a cross between an OXA-resistant (HR) male and an OXA-sensitive (LE) female parasites by infecting hamsters with 500 male and 500 female cercariae larvae of a single genotype. These were derived from snails infected with a single miracidium larva, which reproduce clonally giving rise to 1000s of single sex, single genotype cercariae larvae. Male and female F1 parasites were crossed in the same way and single F2 miracidia were used to infect snails. 130 infected snails, each shedding cercariae of a single recombinant genotype, were used in the pooling experiments. These single genotype cercariae comprised 39 male and 91 female genotypes.

### Pooling of F2 progeny and selection of adult worm pools

Each of the F2 pools used to infect hamsters contained 754 cercariae, representing the 130 recombinant genotypes (39 male and 91 female) (Additional file 2: Figure S2). We balanced the sex ratio by adding 10 representatives of each male genotype and 4 representatives of each female genotype to the pools used for each infection (390 (39 × 10) male cercariae : 364 (91 × 4) female cercariae per pool). Importantly, the F2 pools used to infect each of the six hamsters were identical in their genetic composition, so the impact of OXA-treatment on genome-wide allele frequencies can be directly examined.

Forty F2 females and 16 F2 male adult worms (56 total) survived oxamniquine treatment in the OXA-treatment group (four hamsters). Higher female survival rate following OXA treatment has been observed previously [30]. This represents a global survival rate of 1.86%, relative to the total number of cercariae used in the infections [56/(4 × 754) × 100 = 1.86%]. In the untreated group (two hamsters), 131 females and 147 males (278 total) were recovered, representing 18.44% of the infecting dose [278/(2 × 754) × 100 = 18.44%]. Hence OXA-treatment results in a ~10-fold reduction in survival relative to controls, consistent with strong drug selection.

### Exome capture performance: quality and efficiency

A set of 125,046 120 bp baits was used to capture the *S. mansoni* exome (Additional file 3: File S1) in the parents and the pools of treated and untreated male and female worms. The baits included 124,983 in the nuclear genome and 63 in the mitochondrial genome. They covered 87.52% of the *S. mansoni* exons (59,801 of 68,326 exons) and accounted for 92.18% of the exome length (14,138,142 of 15,336,803 bp) but also included regions surrounding exons (total length: 14,748,899 bp). No baits were designed for capture exons in gene families that could not be unambiguously mapped to a single location in the reference genome. The sequences covered by baits are referred to as the bait regions. Each captured exon was covered by 2 baits.

**Figure 2:**
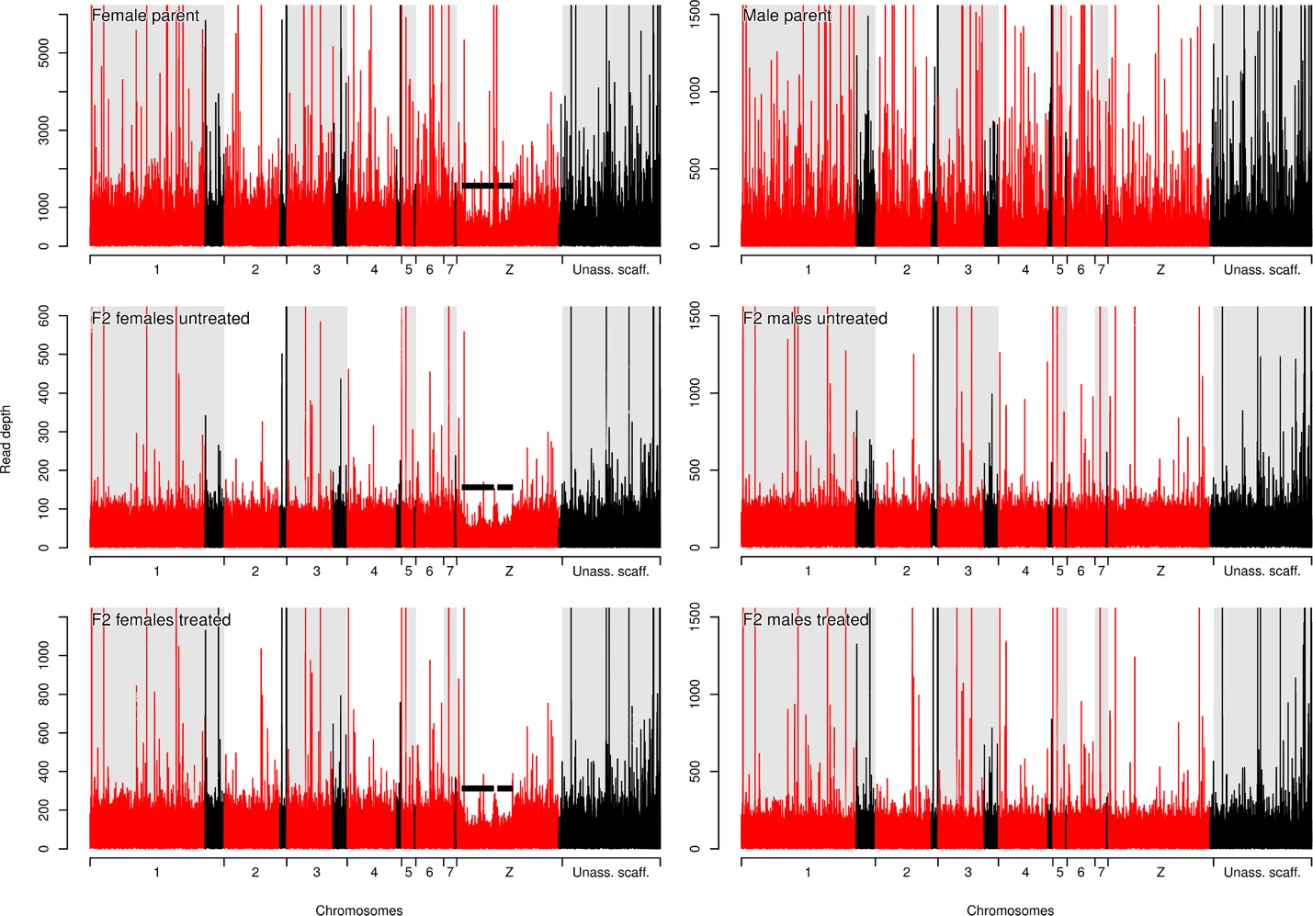
**Read depth of the bait regions for each sequenced library.** Each plot shows read depth of bait regions on the assembled chromosomes (red), on unplaced scaffolds that have been assigned to chromosomes or unassigned scaffolds (black). The black bars show the position of Z-linked regions of the genome, which show approximately 50% reduction in read depth in female (ZW) compared with male (ZZ) worms.

The bait regions had an average read depth of 95 - 366 reads in the 6 libraries. The distribution of the read depth was similar between sexes except for parts of the Z chromosome in the females in which read depth is reduced by half (Table 1, Figure 2). These genome regions (position 3.5-19.5 Mb and 23.5-31 Mb) correspond to the heterochromatin domain of the W chromosome (female sex-specific chromosome) [31] and contain all the Z-specific markers of the genetic map of *S. mansoni* (data not shown) [22].

**Table 1:** **Exome capture library statistics**

			Female parents	Male parents	F2 untreated females	F2 treated females	F2 untreated males	F2 treated males
Total data	Number of mapped reads		87,100,659	25,711,555	21,640,228	31,561,784	39,224,999	29,417,710
Bait regions^1^	Number of reads mapping to bait regions		67,492,249	11,208,843	7,037,441	15,412,158	20,936,397	15,770,592
	Percentage of reads mapping to bait regions		77.49%	43.59%	32.52%	48.83%	53.37%	53.61%
	Read depth	Mean	366	106	95	165	178	125
		Median	274	65	82	133	159	104
Exome	Number of reads mapping to exome		65,114,964	10,837,365	6,808,470	14,939,809	20,271,770	15,311,788
	Percentage of reads mapping to exome		74.76%	42.15%	31.46%	47.33%	51.68%	52.05%
	Read depth	Mean	337	100	90	123	166	117
		Median	250	61	78	104	150	98
Z chromosome	Comparison between Z-linked and non Z-linked regions^2^	Ratio of mean read depth	0.54	1.00	0.54	0.55	1.01	0.97

^1^Bait regions extend beyond the exon boundaries, so the statistics for bait regions and exome are slightly different.

^2^The Z-linked regions are positioned between 3.5-19.5 Mb and 23.5-31 Mb on the Z chromosome.

The statistics for each library indicate efficient exome capture (Table 2). We captured > 99% of the genome covered by the baits in the aligned sequencing data (Table 2). Furthermore, this high capture efficiency was consistent across all six libraries, as well as on each chromosome and on the unassembled scaffold sets.

**Figure 3:**
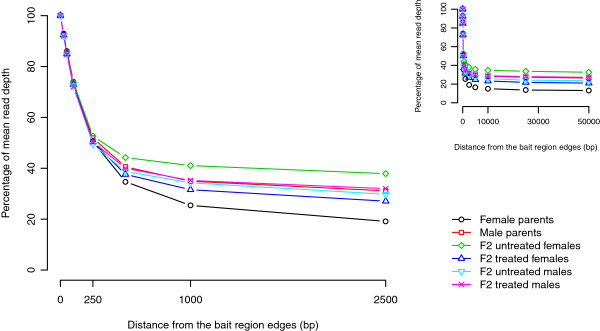
**Mean read depth of the bait regions plus their surrounding regions.** The mean read depth of genome regions adjacent to the baits decreases with the distance from the edge of the bait regions.

The mean sequence read depth declines with distance from the bait regions (Figure 3). Mean read depth was reduced by ~70% at 100 bp, by ~50% at 250 bp, while at 2,500 bp read depth declined to 20-40% of the initial read depth in the 6 libraries. Therefore exome capture provides high read depth sequence for at least 250 bp surrounding bait regions. Inclusion of the 2,500 bp surrounding the bait regions accounted for 60 to 75% of the sequenced data. Taking in account 50,000 bp around the bait regions explained ~97% of the sequenced data. Hence, in addition to the exons, we also captured adjacent genome regions containing promoters, transcription binding sites and other features of interest.

**Table 2:** **Number and proportion of bait coordinates recovered in each library and from each chromosome**

Chromosome	Number of baits	Female parents	Male parents	F2 untreated females	F2 treated females	F2 untreated males	F2 treated males	Mean Total (%)
1	29280	29277	29275	29272	29275	29276	29276	99.98
2	13728	13714	13697	13713	13718	13719	13716	99.89
3	13263	13252	13246	13253	13258	13258	13259	99.93
4	11913	11912	11898	11910	11910	11910	11910	99.96
5	3112	3111	3108	3111	3111	3111	3111	99.95
6	6078	6073	6075	6076	6076	6078	6078	99.97
7	2845	2844	2842	2844	2844	2844	2843	99.95
W	23069	23069	23066	23065	23069	23068	23068	99.99
Unassembled scaffolds	21695	21461	21451	21488	21490	21492	21493	99.01
Mitochondrial	63	63	63	63	63	63	63	100
Total (%)	100	99.78	99.74	99.80	99.81	99.82	99.82

### Variant identification

We identified 14,489 variants in the bait regions of the nuclear genome that were segregating between parents and the F2 progeny. This represents one variant every 1.018 kb on average. 4.10% (600 variants) and 19.91% (2,885 variants) were heterozygous (allele frequency between 0.4 and 0.6) in male parent (HR) and in female parent (LE), respectively, suggesting elevated inbreeding in the male (HR) parent. Among the 14,489 variants, 14,132 were SNPs, 187 were insertions (1-22 bp), and 170 were deletions (1-16 bp).

### X-QTL mapping: oxamniquine resistance QTL identification

**Figure 4:**
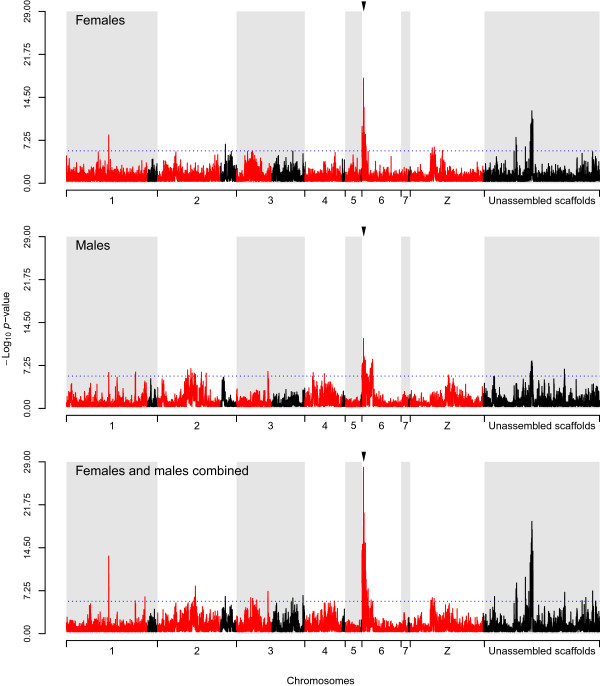
**X-QTL mapping of the oxamniquine resistance QTL.** *p*-values showing the significance of difference in allele frequencies between the treated and untreated pools. In red the region belonging to the assembled chromosomes, in black the region belonging to unplaced scaffolds on the chromosomes or the unassembled scaffolds. The arrow head shows the position of the known SNP of the gene involved in oxamniquine resistance [10]. The dash blue line corresponds to the Bonferroni correction.

We examined the differences in allele frequencies of each variant between treated and untreated worm pools across the exome to identify QTLs involved in the OXA-resistance (Figure 4). To do this we plotted the *p*-values of the *Z*-scores from the allele frequency comparison for both male and female worms at each variant across the bait regions. In addition, we examined the significance of the combined -scores from the comparisons of each sex.

Comparison of treated and untreated female pools shows 72 significant positions, while for treated and untreated male pools 118 positions were significant. Both comparisons reveal strongly significant enrichment of alleles from the resistant parent at the end of chromosome 6. In the female comparison, there were 40 significant positions between positions 227,149 and 4,189,610 (|| ≥ 4.70, *p* ≤ 1.27.10^-6^). Similarly, in the male comparison, there were 81 significant positions (|| ≥ 4.74, *p* ≤ 1.06.10^-6^) between positions 204,937 and 10,866,071. The most significant peak observed in both male and female comparisons was position 1,286,622 ( = 8.69, *p* = 1.86.10^-18^ in females;  = 6.97, *p* = 1.60.10^-12^ in males). This peak is 233.343 kb away from the end of the causative gene (Smp_89320, position 1,519,965-1,524,755) involved in oxamniquine resistance, which was recently identified by conventional linkage methods [10]. We also observed a second major region (unassembled scaffold SC_0154) that shows enrichment of alleles in both male and female comparisons. This scaffold accounts for 22 significant polymorphisms (|| ≥ 4.74, *p* ≤ 1.06.10^-6^) in females and 13 (|| ≥ 4.72, *p* ≤ 1.18.10^-6^) in males.

The remaining significant genome positions (10 in females and 24 in males) were not consistent between the two comparisons. For example, a region on chromosome Z at positions 12,660,165, 15,218,811, 16,201,782, and 23,950,413 (|| ≥ 4.74, *p* ≤ 1.04.10^-6^) accounted for 4 significant peaks only in females, while the other remaining 6 significant positions were found on chromosomes 1 and 2, and within unassigned scaffolds. In males only, a region of chromosome 2 (positions 17,450,959-29,107,158, || ≥ 4.74, *p* ≤ 1.07.10^-6^) shows marginally significant values at 11 variants. The other remaining 13 significant positions were found on chromosomes 1, 3, 4, Z, and within unassigned scaffolds.

Plotting the combined *Z*-scores from the female and male strengthens the signal revealed in both male and female comparisons. Of 166 significant positions genome-wide, chromosome 6 (between positions 55,761 and 10,770,876), accounts for 79 significant positions (|| ≥ 4.74, *p* ≤ 1.08.10^-6^), including the most significant (position 1,286,622,  = 11.07, *p* = 8.74.10^-29^). The unassembled scaffold SC_0154 previously identified in both female and male mapping was confirmed in this combined mapping, with 37 significant positions (|| ≥ 4.72, *p* ≤ 1.20.10^-6^). The other remaining 50 significant positions were found on chromosomes 1, 2, 3, 4, Z, and within unassigned scaffolds.

## Discussion

### Efficiency of exome capture

Capture was extremely efficient since more than 99% of the targeted regions were recovered and sequenced to high read depth in the six libraries (95-366×) on two lanes of an Illumina HiSeq. Furthermore, while the exome constitutes just 4% of the genome, between 32% to 77% of the capture sequences mapped to the bait regions. The exome bait sequences also captured adjacent non-coding DNA, with sequence read depth dropping to 50% of mean exonic read depth 250 bp from the edge of bait regions, as commonly found in exome capture experiments with other organisms [32]. This is an added bonus for functional genomics applications as promoter polymorphisms and other regulatory sequences are often located within 250 bp of coding regions [33]. We also obtained low read depth of sequence from genome regions up to 50 kb distant from bait regions.

Genotyping of modest numbers of microsatellite loci (7-12) is widely used for population genetics of schistosomes [34, 35]. In this study we genotyped 14,489 exonic SNPs (one every 1.018 kb on average) segregating in a single genetic cross. Dense genotyping by exome sequencing will provide a powerful approach for population genomic analysis of schistosome populations. A complication of working with schistosome infections of humans is that adults are found in the blood vessels and only eggs can be collected from infected patients. However, exome sequencing of miracidia hatched from eggs should be possible following whole genome amplification [36].

Analysis of the relative read depth suggests that exome sequencing will be useful for analysis of copy number variation. Males carry two Z chromosomes while females carry only one Z chromosome and a W chromosome [37]. Z and W chromosomes share extensive regions of homology (pseudo-autosomal regions), but there are also Z-linked regions that are present as a single copy in females and two copies in males. The presence of Z-linked regions can be clearly inferred from the read depth plot in the sex chromosomes of males and females (Figure 2). These regions correspond exactly to those identified by Lepesant *et al*. and carrying W specific sequences [31], and also correspond to the positions of markers showing Z-linked inheritance patterns in the cross used to construct the genetic map [22].

### Validation of X-QTL for *Schistosoma mansoni*

**Figure 5:**
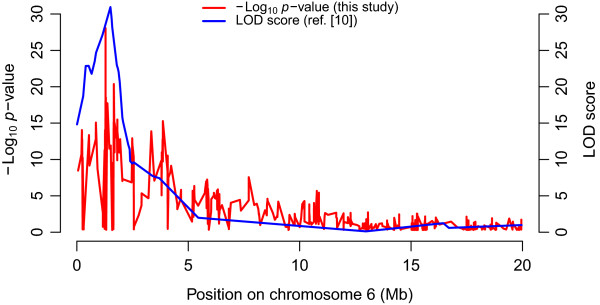
**Comparison of the** ***p*** **-values from the X-QTL mapping and the LOD scores from the classical mapping by Valentim** ***et al.*** **[**[10]**].** The LOD scores are those obtained by the classical linkage mapping previously used to identify the QTL involved in the oxamniquine resistance and are superposed to the *p*-values from the combined  scores used for the present X-QTL mapping.

The X-QTL analysis successfully identified a region of chromosome 6 showing enrichment of alleles from the drug resistant parent in both male and female comparisons. This region contains the causative locus underlying resistance to this recessive trait (Smp_089320) that was identified using classical linkage mapping [10]. The genome position showing the lowest *p*-value was 233 kb (<1 cM) from Smp_089320. This precision is extremely encouraging, given the small number of progeny in the pools. Moreover, when comparing the LOD scores obtained using the classical linkage mapping and the *p*-values obtained from the combined  scores used for X-QTL, the results are extremely similar (Figure 5).

The X-QTL analysis also revealed another region linked to oxamniquine resistance. Both female and male comparisons identified multiple positions (22 in females and 13 in males) on an unassigned scaffold (SC_0154) showing significant peaks. We suggest two possible explanations. First, this scaffold could belong to the end of the chromosome 6, adjacent to the major QTL peak identified. Second, this scaffold could be positioned elsewhere in the genome and identify a second QTL region influencing resistance. We believe that the second explanation is very unlikely because the oxamniquine resistance trait shows a simple recessive inheritance [17] and only a single QTL was identified by classical linkage mapping [10].

The ability of this X-QTL study to correctly map the genome region underlying oxamniquine resistance is particularly remarkable given that two features of our experimental design were suboptimal. First, QTL is most powerful when large numbers of segregating progeny are examined [2]. However, in this experiment just 16 male worms survived drug treatment and were included in the treated male pool, while 40 treated females comprised the treated female pool. Nevertheless, these small sample sizes were sufficient to reveal multiple highly significant variants within the known QTL region on chromosome 6. Second, we used whole genome amplification to generate sufficient amounts of initial DNA for exome capture and library preparation, rather than directly using genomic DNA. While whole genome amplification has been demonstrated to show high fidelity in *S. mansoni* [36], we expected that this procedure would add further experimental noise by reducing the precision of allele frequency estimation. In view of the efficiency of X-QTL to identify QTL regions for a simple Mendelian trait under suboptimal conditions (i.e. using very small numbers of pooled progeny and whole genome amplification), we expect that this approach will be extremely powerful for investigating the basis of more complex traits, when experimental design is optimal (i.e. large numbers of F2 in progeny pools and no whole genome amplification).

We suggest that the small number of weakly significant peaks observed in regions outside the chromosome 6 QTL or scaffold SC_0154 are unlikely to impact OXA resistance. Instead we believe these peaks result from the small size of the population samples from OXA-treated pools. We note that the pooled F2 progeny were constructed using 10 male and 4 female cercariae of each genotype. Hence, while 16 male and 40 female adult worms were recovered from treated hamsters, the actual number of independent genotypes may have been lower than this (as few as 2 male genotypes and 10 female genotypes). Hence, our statistical correction of sampling effects may be insufficiently stringent. We suggest that future applications of X-QTL to schistosomes should focus on increasing the size of pools analyzed to minimize the impact of such sampling effects.

Besides simplifying QTL identification for simple or complex traits, X-QTL may be the only suitable method for mapping some traits, such as host specificity or compatibility. Schistosomes vary in ability to infect populations of host snails, and this variation has been shown to be heritable [19, 38]. While crosses can be made between parasites utilizing different snail hosts, classical QTL mapping is problematic because F2 parasites that fail to develop in a particular host snail cannot be genotyped. This problem can be resolved by X-QTL analysis, because allele frequencies can be compared between emerging parasite pools from the two host snail populations to identify the genome region(s) associated with specific development within each snail.

## Conclusion

In summary, this study extends the use of X-QTL methods to a multicellular parasite, the human blood fluke *S. mansoni,* and demonstrates the utility of this approach for a wide range of multicellular eukaryotes. The approach was validated by remapping a QTL for oxamniquine resistance, which was recently located using classical linkage mapping methods. This method opens the way for future QTL mapping of traits of biomedical or biological significance in schistosomes such as praziquantel resistance, host specificity, and virulence to both snail and vertebrate host. The study also demonstrates that exome capture constitutes a cost effective approach for genotyping in X-QTL studies. For *S. mansoni*, this approach reduced genome size by ~25-fold, thereby reducing sequencing costs, while maximizing read depth and thereby allowing accurate measurement of allele frequency within pooled samples.

## Methods

### Ethics statement

This study was performed in strict accordance with the recommendations in the Guide for the Care and Use of Laboratory Animals of the National Institutes of Health. The protocol was approved by the Institutional Animal Care and Use Committee of the University of Texas Health Science Center at San Antonio (Permit Numbers: 08093X and 11087X).

### *Schistosoma mansoni*populations

The two laboratory maintained populations of *S. mansoni* used in this study are the HR population (oxamniquine resistant) and the LE population (oxamniquine sensitive). Oxamniquine resistance in HR was selected in the laboratory. The HR population was isolated from a Puerto Rican child by Dr. E. Bueding, then transferred to Dr. Donato Cioli at the Laboratory of Cell Biology in Rome and submitted to further drug selection at Dr. Cioli’s laboratory [39]. The LE population was originally isolated from a patient in 1965 in Belo Horizonte, Brazil, and has since been maintained in the laboratory [40]. Laboratory isolates of schistosomes are genetically variable and are generally maintained as outcrossed populations. We therefore used isogenic parasites generated by infecting snails with single miracidia for conducting genetic crosses (see below).

### Genetic cross

We crossed an LE female to a HR male to generate F1 progeny. Subsequently, a male and female from the F1 were crossed to generate F2 progeny (reared to the adult stage).

For each generation, parasites were obtained as follows: Eggs were collected from the liver and hatched under light to obtain miracidia. Snails (*B. glabrata*) were then infected with only one miracidium. Because sex is determined in the zygote (which develops into a miracidium) by a ZW chromosomal system, monomiracidial infections allowed us to be certain that we were using single clonal types (that is, single genetic individuals of the same sex) in the crosses. After 28 days, snails were exposed to light to shed cercariae. Within the snail, schistosomes proliferate clonally, so the emerging cercariae from a snail infected with a single miracidium form a genetically homogeneous population of a single sex. Cercariae were sexed following the protocol described in Criscione *et al*. [22]. Briefly, DNA from cercariae shed by monomiracidial infected snails was extracted using Chelex® and PCR was performed to discriminate between males and females using the W1 primers [41], which are specific to a repetitive region on the W chromosome in females. As this test depends on the failed amplification in males, we performed a concurrent PCR under the same conditions with the autosomal locus sc18 [22] to ensure that the DNA had successfully been extracted from each sample. Upon identification of gender, snails were shed again to collect cercariae for infections. To establish parental and F1 crosses, hamsters (one for each generation) were exposed to 500 female cercariae of a single genotype and 500 male cercariae of a single genotype. For F2 progeny, 390 male cercariae representing 39 genotypes and 364 female cercariae representing 91 genotypes were used to infect each of 6 hamsters (Additional file 2: Figure S2). After 45 days, hamsters were euthanized and perfused to collect adult worms and eggs from the liver for the next generation [42].

The parent worms were separated by sex and pooled for DNA extraction.

### Phenotypic selection

We identified all snails that were infected with F2 schistosomes, and sexed the cercariae larvae emerging. We then made replicate pools of F2 cercariae for infecting hamsters. These pools (i) had identical composition of cercariae from each infected snail (Additional file 2: Figure S2). Hence, changes in allele frequency in control and treated animals at markers should reflect the action of drug selection.

Following maturation, the F2 adult worms were first selected for drug resistance *in vivo*. On day 43, 45, and 48 post-infection, the four F2 infected hamsters in the treatment group were treated by gavage with 50 mg.kg^-1^ oxamniquine solubilized in 2.5% (m/v) Cremophor® EL (Sigma-Aldrich). Two F2 infected hamsters were untreated, receiving only the diluent.

On day 50 of infection, hamsters were euthanized and perfused to collect adult worms [42]. Adult worms were transferred to a petri dish containing DMEM culture media (Dulbecco-Modified Minimum Eagle’s Medium, high glucose (4.5 g.L^-1^), bicarbonate buffered (3.7 g.L^-1^)) supplemented with 20% newborn calf serum, 100 U.mL^-1^ penicillin, 100 μg.mL^-1^ streptomycin, and 0.5 μg.mL^-1^ amphotericin B. Worms perfused from the four treated hamsters were further treated *in vitro* with 500 μg.mL^-1^ oxamniquine solubilized in DMSO (Sigma-Aldrich) for 45 minutes, followed by 3 washes with drug-free medium and incubated at 37°C in an atmosphere of 5% CO_2_ for 12 consecutive days. The *in vitro* treatment was done to ensure that all drug sensitive worms were dead. Adult worms perfused from the two control hamsters were treated in the same way, but exposed only to the diluent. The surviving worms from treated and untreated control group were then separated by sex, counted and pooled for DNA extraction.

### Genomic DNA extraction and whole genome amplification

We prepared DNA from the two parental genotypes and from four pools of F2 progeny (untreated males, oxamniquine treated males, untreated females, oxamniquine treated females). Male and female worms were separated for three reasons: (i) males are bigger than females and contain more DNA, which biases allele frequency measurement in mixed pools, (ii) males and females carry different sex chromosomes (ZZ for males, ZW for females), so separation of males and females allows analysis of sex-linked traits, and (iii) male and female worms serve as internal replication of the experiment. Adult worms from each pool were placed in 200 μL of 6% Chelex® containing 0.2 mg.mL^-1^ of proteinase K, incubated at 56°C for 2 h, and boiled at 100°C for 8 minutes. For each pool, four independent whole genome amplifications (WGA) were performed using the GenomiPhi V2 DNA amplification kit (GE Healthcare) [36] and then pooled to minimize bias from the WGA. DNA was quantified using the Qubit® dsDNA BR assay (Invitrogen).

### Exome capture and sequencing

The *S. mansoni* exome was captured using the SureSelect^XT^ Target Enrichment System (Agilent) according to the manufacturer’s protocol. Three micrograms of WGA DNA were used for each library preparation. The PCR cycles used for the pre-capture and post-capture amplifications were 4 and 12, respectively. The capture was performed using baits (120 bp RNA molecules) specifically designed by Agilent based on the exon annotation of the *S. mansoni* reference genome (gene file (v. 4): ftp://ftp.sanger.ac.uk/pub/pathogens/Schistosoma/mansoni/genome/Gene_models/ARCHIVE/v4.19.05.11.chado.filtered.gff; reference genome (v. 5): ftp://ftp.sanger.ac.uk/pub/pathogens/Schistosoma/mansoni/genome/Assembly-v5/sma_v5.0.chr.fa.gz). The bar coded libraries from the exome capture were sequenced using 100 bp pair-end method on two lanes of a HiSeq 2000 sequencer (Illumina). Raw sequence data has been submitted to the NCBI Sequence Read Archive under accession number SRP033214.

### Sequencing data processing, variant identification and functional effect prediction

The sequencing data were aligned against the *S. mansoni* reference genome (v. 5) using BWA (v. 0.6.1) [43] and SAMtools (v. 0.1.18) [44]. Realignment around indels, Q-score recalibration (using SNPs from [10]), and variant (SNP/indel) calling by the UnifiedGenotyper module were performed using GATK (v. 1.4-37) [45, 46]. Bait representation, capture efficiency and read depth analyses were performed using BEDTools (v. 2.14.3) [47].

The variant calling set was filtered using a minimum Q-score of 40, and a minimum read depth of 10, respectively, in the parental parasites. To refine the calling set, a minimum read depth of 40 was applied for the variants detected in the F2 related libraries. We retained only F2 variants that were segregating in the treated and/or untreated worm pools of each sex.

### Statistical analysis and extreme-QTL mapping

We expect the genome region underlying resistance to be enriched in sequence reads from drug treated F2s. To evaluate statistical evidence for such enrichment, we examined the difference in allele frequencies between treated and untreated F2 pools across the exome. To minimize the bias induced by the unequal number of worms in each treatment and control groups and the difference in the read depth from the different libraries, -scores for each variant were weighted as follows:


where _1_ and _2_ are the estimated allele frequencies in the treated and untreated pools, respectively; _0_ is the allele frequency under the null hypothesis H_0_: _1_ = _2_ estimated from the average of _1_ and _2_; _1_ and _2_ are the number of worms in the treated and untreated pools, respectively, factor  for each  reflecting the ploidy state (=2 except for the haploid Z-linked regions in females where =1); and _1_ and _2_ are the sequencing depths for the treated and untreated pools, respectively.

-scores were computed at each variant site and for the comparison between treated and untreated worm pools of each sex. As worm pools from male and female worms were analyzed separately, -scores were also combined for each variant as follows:


where *Z*_*f*_ and *Z*_*m*_ were -scores from female and male pools, respectively. The *p*-values were obtained by comparing the negative absolute value of *-*scores to the standard normal distribution. To determine the significant threshold, Bonferroni correction was calculated with α = 0.05. These analyses and the graphical representation of the results were conducted using R (v. 2.14.2) [48].

## Electronic supplementary material

Additional file 1: Figure S1: *Schistosoma mansoni* life cycle. The life cycle involves both an aquatic snail intermediate (*Biomphalaria* spp.) and a human definitive host. Rodents can be used to maintain the life cycle in the laboratory. (1) Male (large) and female (thin) adult worms are found in the portal vein. When the worm pairs mate, they migrate into the venules draining the intestine where the females lay eggs which then pass through the intestine wall into the lumen. (2) Eggs leave the body with the feces and hatch in fresh water. (3) Motile miracidia penetrate the intermediate snail host, miracidia differentiate into sporocysts, proliferating asexually. (4) Snails release motile clonal cercariae into the water. (1) Cercariae penetrate the skin of a mammalian host, and migrate through the bloodstream to the hepatic portal system where they develop into adult worms. In the laboratory, the entire life cycle takes 75 to 90 days to complete. *Schistosoma mansoni* is diploid with separate sexes. This aids in the staging of genetic crosses because clonally generated male and female larvae from monomiracidial infected snails can be used to infect mice. (PDF 432 KB)

Additional file 2: Figure S2: F2 adult worm production, selection procedure and pooling strategy. F2 adult worms are obtained by infecting 6 hamsters using identical pools of 390 male cercariae (representing 39 unique genotypes) and 364 female cercariae (representing 91 genotypes). A first *in vivo* drug selection was performed when worms reach maturity within each hamster by treating them with oxamniquine (treated pool) or with diluent only (untreated pool). Worms were then recovered by perfusion, separated by sex and treated again *in vitro*. Surviving worms were finally pooled and DNA extracted. n: number of worms constituting the pool. (PDF 229 KB)

Additional file 3: File S1: Coordinates of the baits used for exome capture across the *S. mansoni* genome. Genomic coordinates of the 125,046 baits used to capture the *S. mansoni* exome. These baits were designed using the version 5 of the genome and the version 4 of the annotation (gff) file. (GZ 853 KB)

## Abbreviations

OXA: Oxamniquine
X-QTL: Extreme QTL
bp: Base pair
cM: CentiMorgan
kb: Kilobases
kg: Kilogram
Mb: Megabase
mg: Milligram
μg: Microgram
μL: Microliter
U: Unit.
