# Supplementary figures and images for: Efficient linkage mapping using exome capture and extreme QTL in schistosome parasites
Source: BMC Genomics. 2014 Jul 21;15(1):617. doi: 10.1186/1471-2164-15-617 (PMC4117968; doi:10.1186/1471-2164-15-617)

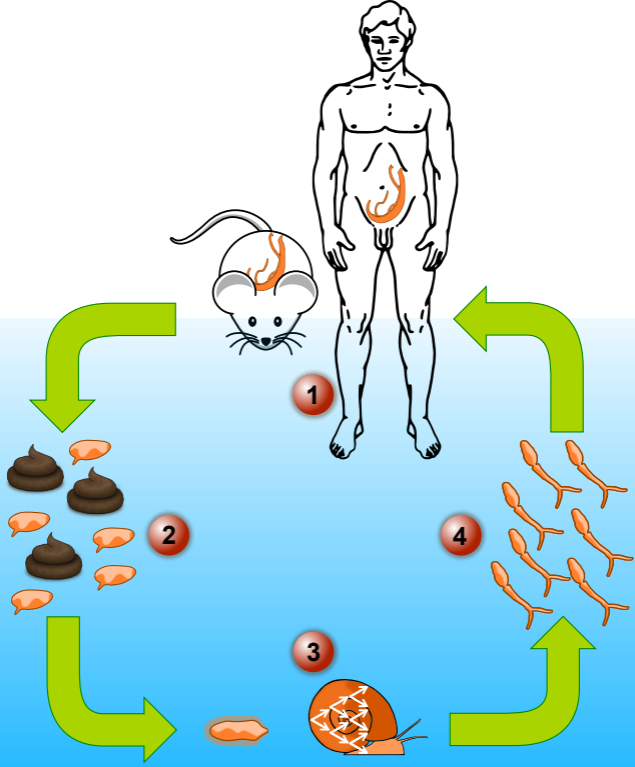

Supplement: Supplementary file 1 — Additional file 1: Figure S1: Schistosoma mansoni life cycle. The life cycle involves both an aquatic snail intermediate (Biomphalaria spp.) and a human definitive host. Rodents can be used to maintain the life cycle in the laboratory. (1) Male (large) and female (thin) adult worms are found in the portal vein. When the worm pairs mate, they migrate into the venules draining the intestine where the females lay eggs which then pass through the intestine wall into the lumen. (2) Eggs leave the body with the feces and hatch in fresh water. (3) Motile miracidia penetrate the intermediate snail host, miracidia differentiate into sporocysts, proliferating asexually. (4) Snails release motile clonal cercariae into the water. (1) Cercariae penetrate the skin of a mammalian host, and migrate through the bloodstream to the hepatic portal system where they develop into adult worms. In the laboratory, the entire life cycle takes 75 to 90 days to complete. Schistosoma mansoni is diploid with separate sexes. This aids in the staging of genetic crosses because clonally generated male and female larvae from monomiracidial infected snails can be used to infect mice. (PDF 432 KB) [file 12864_2014_6296_MOESM1_ESM.pdf]
