# Supplementary material for: Efficient linkage mapping using exome capture and extreme QTL in schistosome parasites
Source: BMC Genomics. 2014 Jul 21;15(1):617. doi: 10.1186/1471-2164-15-617 (PMC4117968; doi:10.1186/1471-2164-15-617)
Supplement: Supplementary file 2 — Additional file 2: Figure S2: F2 adult worm production, selection procedure and pooling strategy. F2 adult worms are obtained by infecting 6 hamsters using identical pools of 390 male cercariae (representing 39 unique genotypes) and 364 female cercariae (representing 91 genotypes). A first in vivo drug selection was performed when worms reach maturity within each hamster by treating them with oxamniquine (treated pool) or with diluent only (untreated pool). Worms were then recovered by perfusion, separated by sex and treated again in vitro. Surviving worms were finally pooled and DNA extracted. n: number of worms constituting the pool. (PDF 229 KB) [file 12864_2014_6296_MOESM2_ESM.pdf]

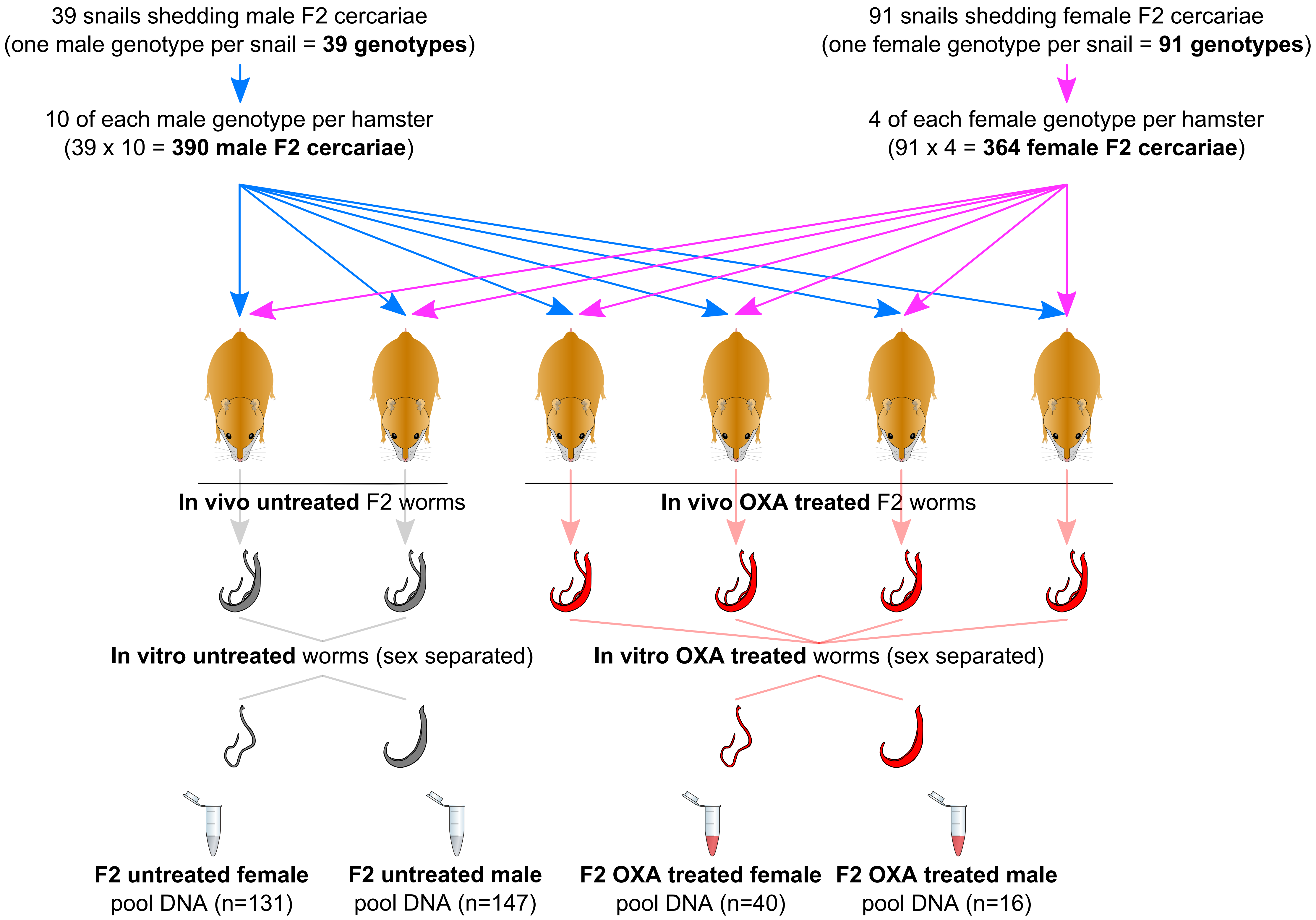
39 snails shedding male F2 cercariae
(one male genotype per snail = **39 genotypes**)
91 snails shedding female F2 cercariae
(one female genotype per snail = **91 genotypes**)
10 of each male genotype per hamster
(39 x 10 = **390 male F2 cercariae**)
4 of each female genotype per hamster
(91 x 4 = **364 female F2 cercariae**)
In vivo untreated F2 worms
In vivo OXA treated F2 worms
In vitro untreated worms (sex separated)
In vitro OXA treated worms (sex separated)
F2 untreated female
pool DNA (n=131)
F2 untreated male
pool DNA (n=147)
F2 OXA treated female
pool DNA (n=40)
F2 OXA treated male
pool DNA (n=16)
